# Socioeconomic inequality in breakfast skipping among Norwegian adolescents

**DOI:** 10.1186/s12937-024-00998-2

**Published:** 2024-08-16

**Authors:** Ingrid Marie Hovdenak, Arnfinn Helleve, Ida Emilie Wolden, Elling Bere

**Affiliations:** 1 Centre for Evaluation of Public Health Measures, Norwegian Institute of Public Health, Oslo, Norway; 2 Department of Physical Health and Aging, Norwegian Institute of Public Health, Oslo, Norway; 3 Department of Sport Science and Physical Education, University of Agder, Kristiansand, Norway

**Keywords:** Adolescents, Breakfast skipping, Socioeconomic status, School

## Abstract

**Background:**

Skipping breakfast is associated with negative health-related and school-related outcomes. Breakfast is the most frequently skipped meal among adolescents. Thus, there is a need to explore the reasons for breakfast skipping across population subgroups to better inform policy makers. The purpose of this study was to present the prevalence of adolescents skipping breakfast on schooldays, analyse the reasons for skipping breakfast and assess associations between the prevalence of skipping breakfast and the reasons for skipping breakfast according to sociodemographic variables.

**Methods:**

The data of a random sample of 10 000 upper secondary school students (aged 16–18 years) from Viken County, Norway, were collected. Students completed a questionnaire measuring breakfast skipping, reasons for skipping breakfast, and sociodemographic variables. Chi-square tests were used to assess differences between the independent groups (family affluence scale (FAS), parental education, and gender) and skipping breakfast and reasons for skipping breakfast.

**Results:**

22% of adolescents reported that they usually skipped breakfast on all schooldays. Skipping breakfast was more prevalent among females, older students, students with lower socioeconomic status and students in vocational education programmes. The difference in breakfast skipping between students with low and high FAS scores was 31% versus 16%, respectively. The most common self-reported reasons for skipping breakfast were time (59%) and not wanting to eat breakfast (48%). Furthermore, 9% reported health issues, and 3% reported economic constraints as a reason for skipping breakfast. Not wanting to eat breakfast was related to a higher FAS score, health issues were more common among girls, and economic constraints were more common among those with low socioeconomic status.

**Conclusions:**

Skipping breakfast was common among Norwegian upper secondary school students. Lack of time and not wanting breakfast were clearly the most cited reasons for skipping breakfast. Health issues and economic constraints were also cited but were less common. The results showed diverging associations between different demographic characteristics and reasons for skipping breakfast. These results are important for developing effective programs to improve diet among adolescents.

## Background

Breakfast is often characterized as “the most important meal” of the day [1]. It fuels the body with nutrients and energy needed throughout the day and it contributes to an overall healthier diet. Adolescents have high nutritional needs due to physical growth and brain development [2]. The intake of a breakfast meal may contribute to these needs. Breakfast habits also likely persist into adulthood [3], and a breakfast meal is often a routine activity that, in the case of children and adolescents, involves social interactions with other household members.

Observational studies among children and adolescents have revealed associations between skipping breakfast and several negative health-related outcomes, such as poorer diet quality in general, greater overweight and obesity, and poorer mental health [1, 4–7]. In addition, skipping breakfast is also associated with school-related outcomes, such as poorer school attendance, poor academic performance and poor classroom behavior [1, 4, 8].

However, these results are mainly associations from observational studies, and experimental studies tend to not confirm these strong associations [9–15]. The dietary benefits of a breakfast meal are not necessarily relevant if nutritional needs are met throughout the day through other meals.

Breakfast is the most frequently skipped meal among adolescents. A systematic review reporting the prevalence of breakfast skipping in 33 countries revealed that 10–30% of children and adolescents did not eat breakfast [7]. Recent results from a Norwegian national survey conducted in 2022 reported that 23% of adolescents in secondary school and upper secondary school usually skip breakfast on weekdays [16].

According to a recent publication from the European Health Behaviour in School-Aged Children Study, the number of daily breakfast consumers (i.e., breakfast every weekday) varied from 38% in Slovenia to 72% in the Netherlands. In this study, 62% of Norwegian 15-year-olds reported eating breakfast every day [17], and in most European countries, the percentage of adolescents (15-year-olds) who ate breakfast every school day decreased between 2002 and 2018. However, it has been stable in a few countries, including Norway, and has increased in a few [17]. Older adolescents attending upper secondary school have higher rates of breakfast skipping than younger age groups (e.g., children in primary and secondary school) [16, 17].

Inequalities are associated with breakfast consumption among adolescents [16–20]; higher socioeconomic status (SES), male gender, living in two-parent families, eating breakfast with one’s parents, and the availability of breakfast food at home have all been associated with regular breakfast intake.

In 2021, the Norwegian government stated that they gradually will implement a school meal program in Norway [21]. The majority of the 350 upper secondary schools in Norway have a school canteen with food for sale, and 45% of these schools already provide a daily free school meal, usually provision of oatmeal porridge before school start [22]. In the Norwegian Action Plan for a Better Diet, one of the goals is to increase the proportion of 15-year-olds who eat breakfast every day [23].

School meal programs have been implemented in several high-income countries [14, 24]. The implementation of universal free meal programs is associated with improved dietary quality and academic performance [14], especially for those with low food security [15]. However, the results are less clear for breakfast than for lunch programs [14], and the context of the conducted studies might not be relevant for the current Norwegian context [25].

Due to the lack of evidence of causality between breakfast skipping and outcomes such as overweight status and academic performance, it remains an open question whether there is potential for improved health and school performance for adolescents who skip breakfast. To estimate the potential effect of school breakfast programs, it is important to know who does not eat breakfast and why they do not eat breakfast.

We do not know why Norwegian adolescents skip breakfast or whether reasons for skipping is associated with sociodemographic factors. In one study from Australia and England from 2014, adolescent breakfast skippers reported reasons for skipping breakfast to be lack of time or being too busy, lack of appetite, not enjoying breakfast, and weight control [26]. However, this was based on answers from only 58 breakfast skippers.

Considering the plan to implement a national school meal program in Norway, there is a need to explore the reasons for breakfast skipping across population subgroups to better inform policy makers and to develop effective strategies for better diets.

Therefore, the purpose of this study was to explore (1) the prevalence of Norwegian adolescents in upper secondary schools in Viken County skipping breakfast on schooldays, (2) the reasons for skipping breakfast and (3) the associations between the prevalence and reasons for skipping breakfast and sociodemographic variables.

## Method

### Data collection and participants

The data used in this study were collected as part of a cross-sectional school meal survey among upper secondary school students (aged 16–18 years) in Viken County conducted in February 2021. In total, approximately 36% (*n* = 16 034) of all upper secondary school students in Viken responded to the questionnaire. In a random sample of 10 000 participants, reasons for skipping breakfast were categorized (see below), and this constituted the present study sample.

All public upper secondary schools in Viken County (*n* = 58) were invited to participate in the study by the educational department in the county. The schools received information and instructions on how to carry out the survey in the classroom during school hours. The students received the link to the web-based questionnaire at Nettskjema run by the University of Oslo. Before the students answered the survey, the teacher, who was present in the classroom, read out load information about the survey.

All data were collected via an electronic, voluntary and anonymous survey. Students gave informed consent by submitting their survey responses (actively pressing ‘send’). The Data Protection Officer at the Norwegian Institute for Public Health undertook a data protection assessment and recommended the project, see https://www.fhi.no/en/ab/this-is-the-norwegian-institute-of-public-health/privacy-policy/.

### Questionnaire

The questionnaire was developed by the Norwegian Institute of Public Health and was pilot tested in collaboration with the municipality of Oslo. The short survey included items on dietary habits during the school day, well-being at school and concentration. In addition, sociodemographic questions such as questions about gender, grade level, school, parental education, economic situation, and the family affluence scale (FAS) were included. Most questions were retrieved from previous surveys among adolescents in Norway [27–29]. The survey included 30 questions and took approximately 10 min to complete.

The question used to assess breakfast habits was *“How often do you usually eat breakfast during the school* week?” The response options were “5 times a week, 4 times a week, 3 times a week, 2 times a week, 1 time a week, I usually don’t eat breakfast”. The last response category was used as a measure of breakfast skipping (i.e. usually never eating breakfast on school days).

Respondents who replied that they usually did not eat breakfast during a school week were asked an additional open-ended question: *“What is your main reason(s) for not eating breakfast?”*. Participants responded to the question using their own words to describe the main reasons. This open-ended question was not included in the survey from the beginning, but 8330 (of the study sample om 10 000) had this possibility.

These answers were first coded into 13 categories and thereafter grouped into four main categories (Table 1): *lack of time* (no time, prioritizing, long commute to school), *not wanting breakfast* (not hungry, do not feel for it, cannot stand it, lack of appetite, habit, no desirable food at home), *health-related issues* (nauseous, dieting, eating disorder) and *economic constraints* (poor economy, no food at home).

**Table 1:** Categorization of self-reported reasons for skipping breakfast (main and subcategories and examples)

Main categories	Subcategories	Examples
Lack of time	No time	“Don’t have time in the morning”
	Prioritizing	“Prioritize sleep over food”
	Long commute to school	“Don’t have time due to long distance to school”
Do not want breakfast	Not hungry	“Not hungry in the morning”
	Do not feel for it	“Don’t bother”
	Cannot stand it	“Can’t bear it”
	Lack of appetite	“No appetite”
	Habit	“Not a breakfast person”
	No desirable food at home	“No food at home that tempts me”
Health related issues	Nauseous	“I am nauseous in the mornings”
	Dieting	“Trying to lose weight”
	Eating disorder	“Having eating disorders”
Economy constraints	Poor economy	“Can’t afford”
	No food at home	“No food at home”

The family affluence scale (FAS) is a measure of SES regarding material affluence (economic capital) by the characteristics of the household [27]. It consists of four questions: *Does your family own a car?* (No = 0; Yes, one = 1; Yes, two or more = 2), *Do you have your own bedroom?* (yes = 1; no = 0), *During the past 12 months*,* how many times did you travel away on holiday with your family?* (Not at all = 0; One = 1; Two = 2; More than twice = 3), *How many computers do your family own?* (Non = 0; One = 1; Two = 2; More than two = 3).

The scores of the four variables were summarized. The score ranged from 0 (lowest affluence) to 9 (highest affluence). The ordered score was trichotomized and coded as low, medium or high.

Parental education, as a measure of SES, was assessed by the following question: *Do any of your parents have higher education?* (No; yes, one; yes, both). The answers were kept as a trichotomous variable.

Demographic characteristics in this study included study programme (vocational or specialization in general studies, gender and grade). The gender categories included males and females (the options “other/do not want to respond” were not included in the analyses). The grades included (1) grade, (2) grade, and (3) grade (the option “not relevant” was not included in the analyses).

### Analyses

Chi-square tests were used to assess differences between the independent variables (gender, grade, FAS, parental education, and field of study) and skipping breakfast (Table 2) and the reasons for skipping breakfast (lack of time, not wanting, health issues and economic reasons) (Table 3). Significance level was set to 0.05. All analyses were conducted in STATA.

## Results

Overall, 22% of the adolescents reported that they usually skipped breakfast on all schooldays. Skipping breakfast was more prevalent among females than among males, adolescents in higher vs. lower grades, those with lower FASs than among those with higher FASs, those with parents without higher education than among those with parents with higher education, and among students at vocational education programmes than among those with students at education programmes for specialization in general studies. The difference in breakfast skipping between students with low and high FAS scores was substantial (31% versus 16%) (see Table 2).

**Table 2:** Breakfast skipping (%, n) by gender, grade, FAS, parental education, and study programme among upper secondary school students in the county of Viken, 2021 (total study sample; *n* = 10 000)

	Categories	*n*	Skipping breakfast (%)	*p* value
Gender	Male	4353	20	< 0.001
	Female	5445	24	
Grade	1.grade	4065	21	< 0.001
	2.grade	3498	22	
	3.grade	2395	25	
FAS-categorical	Low	2031	31	< 0.001
	Medium	4645	23	
	High	3180	16	
Higher parental edu.	No	1654	31	< 0.001
	Yes, one	2597	22	
	Yes, both	4237	17	
Study programme	Vocational education	3000	28	< 0.001
	Specialization in General Studies	6945	20	
Total		9958	22	

p values from Chi-square tests

A total of 1765 students reported their main reason(s) for skipping breakfast in the open question. The respondents could state several reasons. The most common self-reported reasons for skipping breakfast were lack of time (59%) and not wanting to eat breakfast (48%). Furthermore, 9% reported health-related issues, and 3% reported economic constraints as reasons for skipping breakfast.

Table 3 presents the associations between demographic characteristics and reasons for skipping breakfast. A greater percentage of females (12%) than males (4%) reported health-related issues related to skipping breakfast. There were no significant associations between gender and other factors.

With regard to socioeconomic status, there was a significant association between the FAS and the reasons for not wanting breakfast and economic constraints. A greater percentage of adolescents with a high FAS (53%) than with a medium FAS (49%) or low FAS (42%) reported not wanting breakfast as a reason for skipping breakfast. Furthermore, a greater proportion of adolescents with a low FAS (7%) reported economic constraints as a reason for skipping breakfast than did adolescents who scored medium (1%) or high (1%) on the FAS scale. No significant association was found between the FAS score and time- or health-related issues.

There was a significant association between higher parental education and economic constraints. A greater percentage (6%) of adolescents with no parents with higher education reported economic constraints as a reason for skipping breakfast than did those with one (2%) or two (1%) parents with higher education. No other significant associations were observed for parental education.

**Table 3:** Reasons for skipping breakfast (%), by gender, FAS, and parental education (*n* = 1765)

		Lack of time %	*P* value	Do not want breakfast %	*P* value	Health related issues %	*P* value	Economy constraints %	*P* value
Gender	Male	58	0.416	49	0.460	4	< 0.001	3	0.298
	Female	60		47		12		2	
FAS	Low	62	0.184	42	0.002	7	0.174	7	< 0.001
	Medium	59		49		9		1	
	High	56		53		10		1	
Higher parental education	No	57	0.391	47	0.948	8	0.834	6	< 0.001
	Yes, one	60		47		9		2	
	Yes, two	62		46		9		1	
TOTAL		59		48		9		3	

p values from Chi-square tests

## Discussion

This study investigated the prevalence of breakfast skipping among upper secondary school students in the Norwegian county of Viken. The results revealed that 22% of the students typically skip breakfast on all school days, which aligns with the findings of other studies nationally [16, 29] and internationally [7, 17]. The findings suggest that a substantial proportion of youth do not eat breakfast on schooldays. While there are a range of perceived potential benefits to eating breakfast, it is still not necessarily a goal that every youth should eat breakfast on a daily basis. From a public health perspective, the objective should be to ensure that any schoolchild who desires breakfast has access to it.

This study revealed substantial differences in skipping breakfast based on socioeconomic status, both according to family affluence (31% among students with low SES versus 16% among those with high SES) and parental education (31% among students with parents lacking higher education versus 17% among those with both parents holding higher education degrees). Additionally, breakfast skipping was more prevalent among females and older adolescents, consistent with previous research [7, 30, 31].

The significantly greater proportion of breakfast skipping among students with lower socioeconomic status raises the question of whether these differences stem from structural drivers or from individual choices and preferences.

When the study participants provided their reasons for skipping breakfast, the predominant factors cited were lack of time (58%) and not wanting breakfast (49%). Furthermore, a smaller proportion mentioned health-related issues (9%) and economic constraints (3%). Few comparable studies exist, but our findings are consistent with those of a study from 2014 among 14-15-year-olds from Australia and England, where the most frequently cited reasons included not having time or being too busy (43%), not being hungry in the morning (24%), not enjoying breakfast (16%), and weight control (4%) [26]. Older studies also confirm that lack of time and not wanting breakfast are the two major reasons for skipping breakfast, with some adolescents reporting health-related issues such as weight control and very few reporting no access to food at home [26, 32–34].

The students’ self-reported reasons for skipping breakfast revealed that there was no association between lack of time and socioeconomic status or gender. However, significant associations with SES emerged between those who said they did not want breakfast and those who referred to economic constraints. Interestingly, higher SES was associated with a greater likelihood of not wanting breakfast, while attributing breakfast skipping to economic constraints was significantly associated with low SES.

In essence, the socioeconomic disparity in breakfast skipping was more pronounced in their reports of breakfast skipping (Table 2) than in their self-reported reasons (Table 3). One plausible explanation for this difference could be linked to their perceptions of social acceptability in their open-ended self-reports. Declaring that they skipped breakfast due to personal preference represents an assertion of individual choice, while attributing it to poor household economy might be considered stigmatizing and even shameful. In 2020, a total of 115 000 children and adolescents (ca. 11%) in Norway lived in a household with persistently low household income [35]; therefore, it is plausible that some upper secondary school students do not eat breakfast because of poor household income. With increasing inflation and food prices, the proportion may have increased since these data were collected [36].

Even if there are more structural reasons why uppers secondary school students skip breakfast, most of the reasons given by the youth themselves were at an individual level. In our sample, the most common reason for skipping breakfast was lack of time (59%). The adolescents mentioned, for instance, prioritizing sleeping longer and long commutes to school as reasons for skipping breakfast under this category. It has furthermore been reported that adolescents have to wake up earlier than their “natural” wake-up time and may feel tired on school days [37, 38]. A systematic review study of skipping meals among young adults (18–30 years old) also revealed that time or lack of time was the strongest correlate of meal skipping [39]. Our finding that time constraints are also a major reason for adolescents not eating breakfast therefore seems reasonable.

The other main reason for skipping breakfast reported in the sample was not wanting breakfast (48%). Examples of reasons include lack of appetite and not being hungry in the morning. There might be different biological reasons for not being hungry in the morning. The nightly fasting state, the body’s internal clock, or circadian rhythm, and other individual metabolic variabilities might explain why some adolescents are not hungry in the morning [40]. Sleep quantity and quality might also impact morning appetite and breakfast consumption, and studies have reported that sleep duration and morning tiredness are both independently correlated with daily breakfast consumption among adolescents [37, 41]. Another potential reason is dietary habits, and findings suggest that higher caloric intake at dinner and in the evening are linked to a greater likelihood of skipping breakfast [42]. Breakfast might not be “the most important meal”.

Finally, health issues were referred to as a reason for skipping breakfast, notably more among girls than boys (12% versus 4%). Health issues included responses such as dieting and eating disorders, which are more common in females than in males [43, 44]. The gender differences may indicate greater body image concerns and dieting concerns among females than among males. Conversely, there were no clear gender differences for other reasons for skipping breakfast. Fasting was mentioned as a reason by a few, and during recent years, intermittent fasting, which is a short-term fast (e.g., extending the night fast by not eating breakfast) to improve body composition and overall health [45], has gained increased attention. It is possible that some adolescents have caught up on this trend, but this should be further investigated.

### Strengths and limitations

A key strength of this study is that it explores the reasons for breakfast skipping across population subgroups. This information is important for developing and/or improving strategies for better diets, e.g., school breakfast programmes. We included data from a large sample (10 000) representative of the largest Norwegian County (a county that, in 2024, is split into three smaller counties; Akershus, Buskerud and Østfold), including 1765 breakfast skippers reporting qualitative data on reasons for skipping breakfast. This is also the first study to assess sociodemographics associated with different reasons for skipping breakfast in the international literature, as far as we know.

A limitation of the current study is the cross-sectional design, which precludes any causal interpretations of the results. The questions in the FAS-score might appear strange, considering the context of technological, ecological and social change. Also, the FAS-score and parental education is reported by the students and not by the parents themselves. However, the FAS score has been reported to be useful for ranking affluence and measuring health outcomes at single time points [46]. Furthermore, this study lacked information on breakfast content. Therefore, we are unable to explore the dietary quality of breakfast. It is important to note that a larger percentage of students, in addition to those who skip breakfast, may benefit from a healthy school meal program. In our questionnaire, breakfast was not defined and could be interpreted differently. Also, only those reporting usually not to eat breakfast on school days were defined as breakfast skippers. This gives a clear cut-off, and it is easier to interpret, but those skipping breakfast occasionally is not included as breakfast skippers.

In 2022, the proportion of the population with higher education in Viken was similar to the general proportion in Norway (31.8% vs. 31.9%) [47]. The current study sample had a participation rate of 36%, and 80% of the students reported that one or both parents had higher education. Vulnerable groups, i.e., students from poor families with food insecurity, might be underrepresented, and the number of students without food for breakfast at home might in reality be greater.

### Implications

This study revealed that there are subgroups in Norway that would likely eat breakfast more often if school breakfast programs were implemented, especially those few reporting family economy constraints as a reason for not eating breakfast and those reporting a lack of time.

However, the international literature on the effects of school breakfast programs shows mixed results [14], and the context of these studies is not necessarily relevant for the Norwegian context in 2024 [24]. In general, studies have reported that it is a challenge to promote attendance at school breakfast programs [14]. In a recent Norwegian study, few participants attended school breakfast programs [48].

In addition, the benefits of eating breakfast are mainly based on observational data with little causal evidence [9], and several people state that they do not want breakfast.

Therefore, some might be better off not eating breakfast, and programs therefore need to be developed by care.

### Future studies

Little research has been conducted on adolescents’ reasons for skipping breakfast. The current study revealed diverging associations with sociodemographic characteristics for different reasons. Some adolescents might not be hungry before lunch, while others want breakfast but have no options at home. More research is needed to establish how to best organize school meal programs, breakfast or lunch, universally or in a targeted manner.

## Conclusion

In conclusion, 22% of the adolescents in our sample usually skipped breakfast on their schooldays. Skipping breakfast was more prevalent among females, older adolescents, and those with lower socioeconomic status. The most common reasons among those who skipped breakfast were lack of time (59%) and not wanting breakfast (48%), while skipping breakfast due to health-related issues (9%) and economic constraints (3%) were less common. The results showed diverging associations between different demographic characteristics and reasons for skipping breakfast.

## Data Availability

The data that support the findings of this study are available from the corresponding author upon reasonable request.
